# Best Match: New relevance search for PubMed

**DOI:** 10.1371/journal.pbio.2005343

**Published:** 2018-08-28

**Authors:** Nicolas Fiorini, Kathi Canese, Grisha Starchenko, Evgeny Kireev, Won Kim, Vadim Miller, Maxim Osipov, Michael Kholodov, Rafis Ismagilov, Sunil Mohan, James Ostell, Zhiyong Lu

**Affiliations:** National Center for Biotechnology Information (NCBI), National Library of Medicine (NLM), National Institutes of Health (NIH), Bethesda, Maryland, United States of America

## Abstract

PubMed is a free search engine for biomedical literature accessed by millions of users from around the world each day. With the rapid growth of biomedical literature—about two articles are added every minute on average—finding and retrieving the most relevant papers for a given query is increasingly challenging. We present Best Match, a new relevance search algorithm for PubMed that leverages the intelligence of our users and cutting-edge machine-learning technology as an alternative to the traditional date sort order. The Best Match algorithm is trained with past user searches with dozens of relevance-ranking signals (factors), the most important being the past usage of an article, publication date, relevance score, and type of article. This new algorithm demonstrates state-of-the-art retrieval performance in benchmarking experiments as well as an improved user experience in real-world testing (over 20% increase in user click-through rate). Since its deployment in June 2017, we have observed a significant increase (60%) in PubMed searches with relevance sort order: it now assists millions of PubMed searches each week. In this work, we hope to increase the awareness and transparency of this new relevance sort option for PubMed users, enabling them to retrieve information more effectively.

## Introduction

PubMed (www.pubmed.gov) is a widely used search engine, built and maintained by the United States National Center for Biotechnology Information (NCBI) at the US National Library of Medicine (NLM), that provides access to more than 28 million scholarly publications in biomedicine. On an average working day, there are about 2.5 million PubMed users conducting 3 million searches and 9 million page views. Every article and its associated data elements (also known as Fields, such as title, abstract, author names, and author affiliations; see S1 Glossary for definitions and abbreviations) must be first built into the search index of PubMed before users can search. Then, at query time, PubMed employs all the terms specified in the search to find matches in all possible fields. Next, by default, all matching articles will be returned in reverse chronological order. That is, newly published articles are always returned first. While this sort order is desirable for seeking the latest information on a given topic or for an individual author, it may not be ideal for other types of searches (e.g., new topics) or deliver the most relevant articles to our users most efficiently, as irrelevant results can be returned at the top due to query ambiguity and complexity. E.g., if a search intent was to find articles studying a given disease in a certain geographic area or ethnic group (e.g., "melioidosis Taiwan"), then top results matching the location term in the author affiliation field (instead of treating it as a content keyword) would be unsatisfactory. Inability to locate semantic concepts in relative proximity can also result in suboptimal results [1]. The query "cancer related fatigue," for instance, returns many seemingly irrelevant articles on the first page when sorted by publication date.

We have previously observed that over 80% of the user clicks of search results happened on the first page. This user behavior [2] is highly similar to that of general web searches despite the very different date sort order used in PubMed. Thus, for the majority of the PubMed queries for which there are over 20 results, more useful and often still recent papers on page two and beyond could be easily missed by users.

In response, in 2013, a relevance sort option was made available in PubMed that implemented term frequency–inverse document frequency (TF–IDF) weighting, a classic information retrieval (IR) strategy for computing query-document relevancy [3] based on how many search terms are found, in which fields they are found, and the frequency of the term across all documents. Additionally, recently published articles are given an artificial boost for sorting. For databases other than PubMed, alternative IR methods such as BM25 [4] and variations of the classic TF–IDF algorithm have been studied and applied elsewhere [5–9].

While the classic TF–IDF method shows good performance for relevance ranking, all of its parameters (e.g., recency boost factor) are based on manual experiments or analyses. Often, with this approach, parameters are tuned empirically and/or based on domain knowledge. Recent studies have shown that one can build more robust ranking models trained on large-scale datasets by using machine-learning algorithms [10]. Particularly, learning to rank (L2R), a class of machine-learning algorithms for ranking problems, have emerged since the late 2000s and shown significant improvements in retrieval quality over traditional relevance models by taking advantage of big datasets [11]. With a pretrained L2R model, a relevance score is assigned for each matching document given a query, with more relevant documents receiving a higher score. Because of their superior performance, these L2R algorithms have also been recently applied to many other tasks in biomedical research [12–16].

While there are a number of research studies on L2R, few have explored its applicability and feasibility as an end-to-end system for real-world use in biomedicine [17]. Furthermore, although machine-learning or L2R methods have been implemented in large-scale commercial search systems [18], because of proprietary information, little has been published regarding its scalability and overall performance with real users.

To this end, we describe the use of L2R to create a new relevance search algorithm for PubMed search, the first of its kind in (biomedical) scientific literature retrieval to the best of our knowledge. For validating our method, we present both the offline evaluation results (computer-ranked results against a gold standard) as well as the online results when tested with real PubMed users (measured by user click-through rate—CTR). Finally, to demonstrate its utility, we report its usage rate since its full deployment in PubMed in June 2017 with a focus on when and how to use it in practice. In doing so, we hope to increase the transparency of this new relevance sort option (labeled as Best Match in PubMed) for our users such that they can better understand and ultimately search more effectively in PubMed. The technical details (in Supporting Information) may also be beneficial to those who are interested in implementing such a method in production systems. The research source code is available at https://github.com/ncbi-nlp/PubMed-Best-Match.

## Tool description

### Two-stage ranking architecture with improved performance

**Fig 1:**
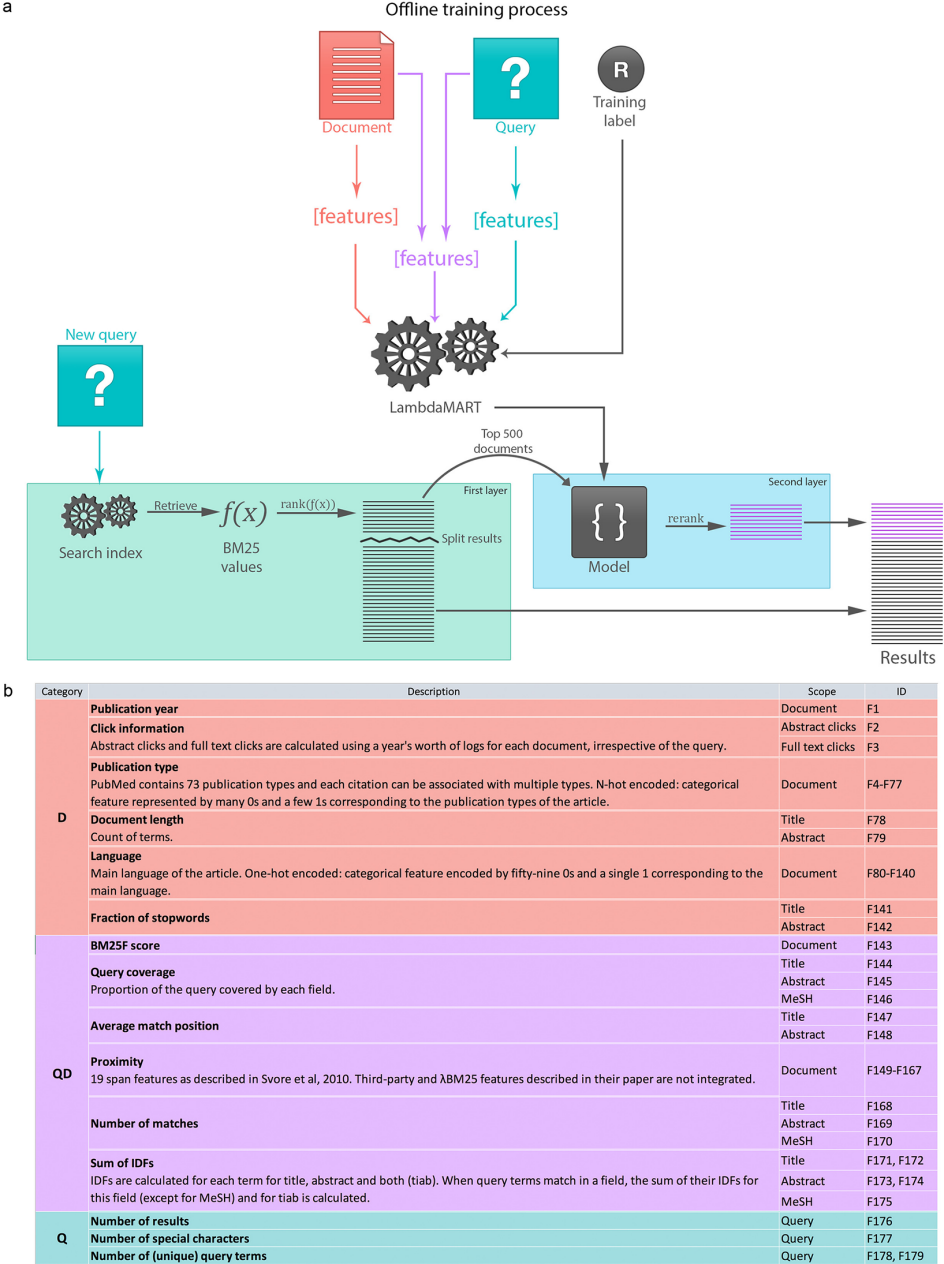
The overall architecture of the new relevance search algorithm in PubMed. (a) It consists of two stages: processing first by BM25, a classic term-weighting algorithm; the top 500 results are then re-ranked by LambdaMART, a high-performance L2R algorithm. The machine-learning–based ranking model is learned offline using relevance-ranked training data together with a set of features extracted from queries, documents, or both. (b) Features designed and experimented in this study with their brief descriptions and identifiers. D, document; IDF, inverse document frequency; L2R, learning to rank; Q, query; QD, query–document relationship; TIAB, title and abstract

For PubMed's Best Match, we adopted a two-stage ranking architecture—in which the two separate steps, retrieval and reordering, can be optimized independently—for using L2R [19,20], as it provides both efficiency and flexibility. As shown in Fig 1A, (1) given a user query translated and mapped to fields automatically, PubMed first retrieves documents that match it and orders them with a classical term weighting function, BM25 (see S1 Text). (2) The top-ranked documents are further sorted by a second ranker called LambdaMART [21] (see S2 Text), which stands out as a robust and fast approach with superlative performance in various ranking tasks (e.g., the 2011 L2R challenge [22] or various TREC tasks [23]). Note that the first layer is very similar to the previous relevance system used in PubMed starting in 2013. The main novelty is thus the addition of the second, machine-learning–based layer.

In order to train LambdaMART and test its effectiveness, a set of gold-standard query-document pairs is required. Given the lack of real-world datasets for biomedical information retrieval, we used the user-click information from PubMed search logs (see S3 Text) as the (pseudo-)gold standard for document relevance and created a benchmark dataset, which contains 46,000 unique queries in total (see S4 Text). A random split of 70% was used for training the LambdaMART algorithm. When evaluated on the held-out test data (the remaining 30%) using the Normalized Discounted Cumulative Gain (NDCG), a standard measure for ranking quality (see S5 Text), our results show that the second ranker is able to learn from the "ground truth" and obtain more than 3-fold increases in ranking quality when compared with the previous TF–IDF method (0.48 versus 0.15 in NDCG scores) (see S6 Text).

### Document ranking features and their impact on performance

Besides labeled data, another prerequisite for training machine-learning algorithms is transforming each data instance into feature representations. Hence, for distinguishing relevant versus irrelevant articles, we designed a set of distinctive features ("ranking factors/signals") that aim to capture the various characteristics of a document D (e.g., publication year or type), the relationship between a query and document QD (e.g., number of query term matches in title), or the specifications of the query Q (e.g., query length). See Fig 1B for a complete list and how they are encoded in S7 Text. Document features are used to represent the inherent nature of documents irrespective of the query. Specifically, we characterize a document in multiple dimensions such as its publication time, publication type, past usage, etc. We use publication year, as we know recency is a critical factor in finding and reading scholarly articles. Similarly, the type of publication can also be important (e.g., review articles are generally desired in a literature survey process). The past usage of an article can be seen as an approximation for assessing its popularity among users. Finally, we also include features such as document length and language for a fuller description of a document.

Query–document features intend to capture to what degree a document is related to the query. For instance, the BM25 score is used as a feature to capture this relationship. We also take into count the number of term matches in specific fields (e.g., title), as well as text proximity—how close the matches are to each other in the document. The latter is used to favor documents in which matched term positions are grouped together rather than scattered over the document. Specifically, we followed the lead of [24] and used 19 features to represent this (e.g., count of words between query terms).

The third group regards queries only, ranging from its length (the number of search terms) to the count of special characters (e.g., those in chemical names) to the number of returned results (as a measure for whether it relates to a broad versus narrow topic).

To assess the importance of each feature (group) towards the overall performance, we conducted feature-ablation studies in which we recorded performance loss when individual (or groups of) features were removed. We find that the D features (especially publication year and past usage) and QD features (especially BM25 relevance score) are the most critical and complementary to each other. Although Q features have a relatively minor effect, they can also contribute to improve the overall ranking quality (see S8 Text).

### Improved search experience in online evaluation with real users

Given the benchmarking results and feature analysis, we proceeded with a widely used web analytics method called A/B testing [25], which compares two or more variations of a feature with real users in a controlled experiment. In our case, for all queries for which users selected relevance sort order, we routed 25% of them to the newly proposed Best Match algorithm while keeping the rest of the queries (75% of total) with the original TF–IDF algorithm. We then compared the CTRs, the fraction of queries with at least one user click on the top-ranked results (see S5 Text). Note that queries for which PubMed returned zero or a single article were excluded from this experiment, as they were not applicable (no click was needed). In addition to focusing on the rank of 20 (the default number of returned results in the first page), we compared CTR@10, CTR@5, and CTR@3 to get a sense of the improvement at top-ranked results. Also, for comparison, we included the results using the default date sort option. This experiment ran from March 1st, 2017, to June 8th, 2017, consisting of 133,822,362 searches by date, 7,527,507 searches routed to TF–IDF, and 2,509,169 searches routed to Best Match.

As shown in Table 1, the new Best Match algorithm performs significantly better than both the default date sort as well as the previous relevance search algorithm at every rank position. Furthermore, relative improvements in CTRs increase steadily as the rank threshold decreases (e.g., 40% improvement for CTR@3 versus 22% for CTR@20 in comparison with date sort results), demonstrating that Best Match is especially better at optimizing the top-ranked results. We also observed that the increase in CTR is applicable to a wide variety of different queries. That is, both popular and infrequent queries benefit from the new Best Match algorithm (see details in S1 Fig). For instance, over 87% of PubMed queries are unique, and they have an average CTR@20 of 0.408—see the GitHub repository for more details.

**Table 1:** Comparison of the user click-through rate of best match versus the previous TF–IDF method and the default date sort order.

Ranking Method	CTR@20	CTR@10	CTR@5	CTR@3
Sort by date	0.32	0.29	0.24	0.20
Sort by TF–IDF	0.36	0.33	0.29	0.25
Sort by Best Match	**0.39**	**0.36**	**0.32**	**0.28**

All improvements in CTRs by Best Match are statistically significant with 99% confidence (paired *t* test). **Abbreviations:** CTR, click-through rate; TF–IDF, term frequency–inverse document frequency.

Note that while the absolute increase in CTRs may seem modest, a relative improvement of 1–2% in CTRs in real-world settings (e.g., online ads seen in web search results) is typically considered successful [26,27]. We also noticed that algorithmic improvements in NDCG scores can translate into more modest real-world improvements in CTR scores. We believe this is due to the fact that search quality is just one of the factors affecting CTRs. E.g., a system that highlights matching terms or returns with snippets (highlights from the article that are related to the user query) would usually have a higher CTR compared to the same results without such visual cues.

### Increased usage of relevance search in PubMed

**Fig 2:**
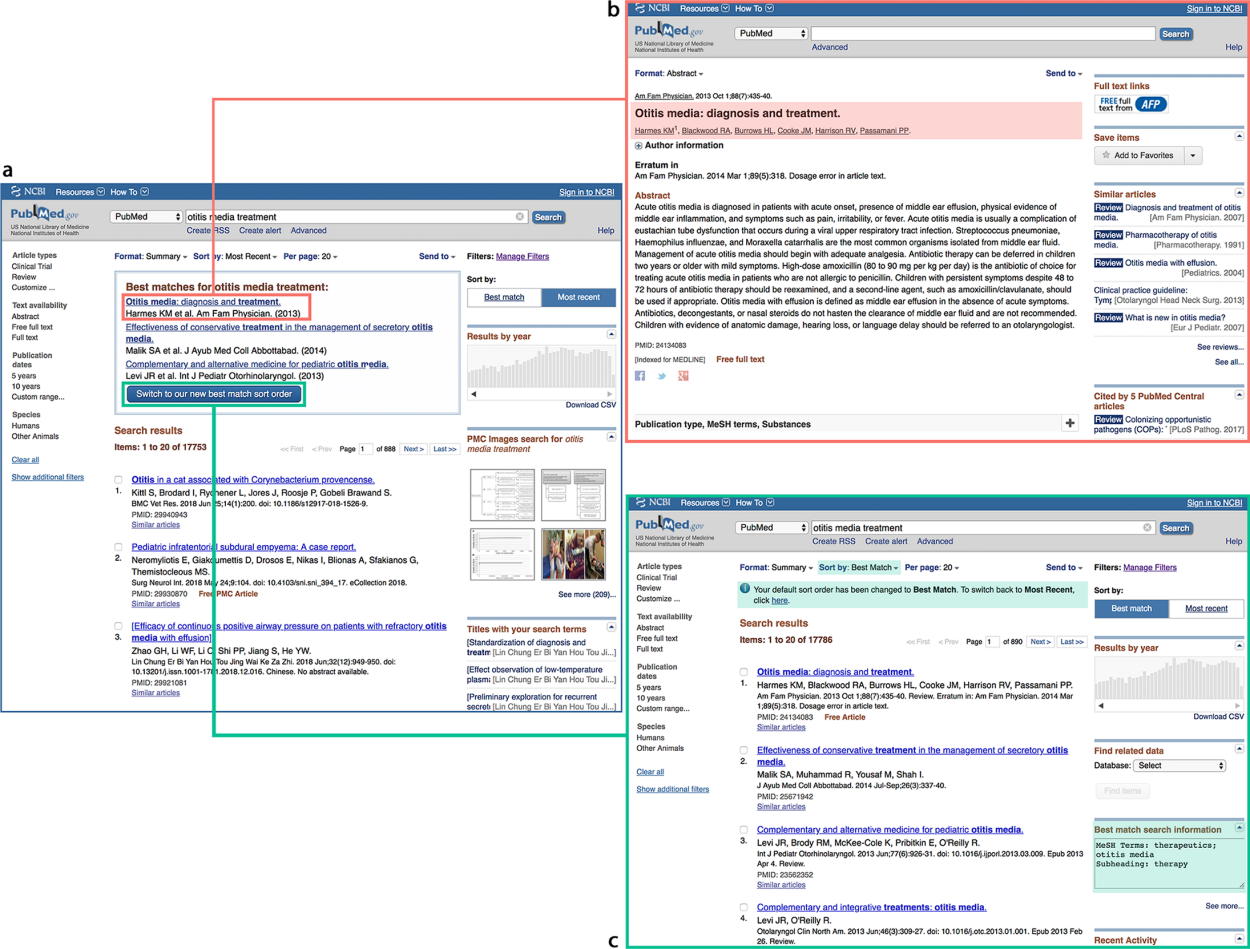
The Best Match search option in action. When our system detects that search results by Best Match could be helpful to our users, a Best Match banner is displayed on top of the regular search results (a). A user can click title(s) to view the article abstract (as shown in (b)) or click on the Switch button see complete results returned by Best Match (as shown in (c)).

Given the significant increase in performance of the new Best Match algorithm over the previous method, we deployed the new algorithm to production in June 2017. To further promote the update, a Best Match banner was developed as shown in Fig 2. Through log analysis during December 2017, we find that the Best Match banners are clicked 1 out of 10 times when displayed, with a much higher chance of follow-up document clicks: CTR@20 of 52% for over 100,000 queries re-run under Best Match after switching the sort order. This is markedly higher than the usual CTR of 39% shown in Table 1. In addition, only a very small percentage (2.5%) of users chose to switch back to the date sort order.

**Fig 3:**
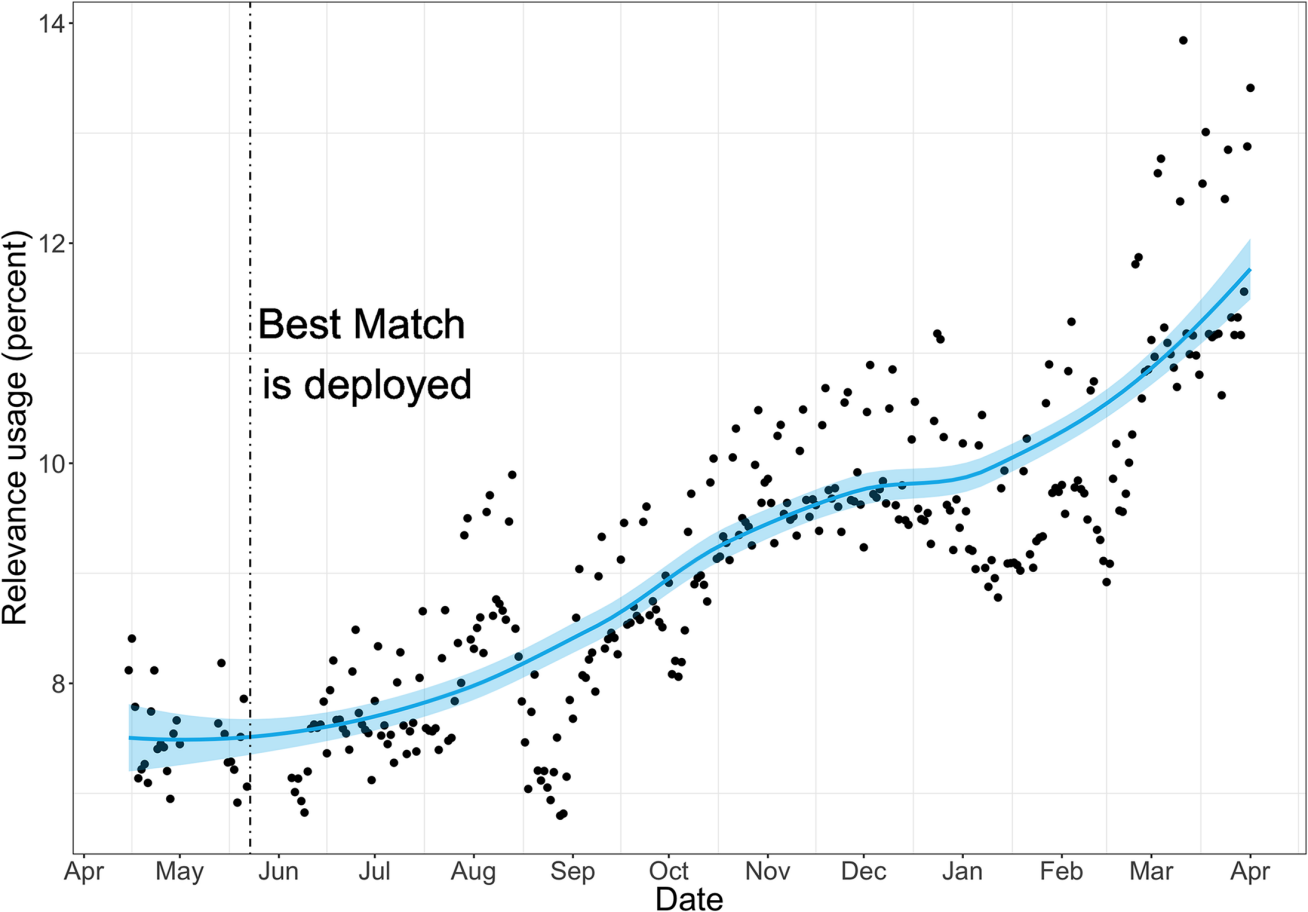
Usage rate of relevance sort order over 6 months (May 2017 to October 2017). The blue line represents the trend, and the blue area represents the variance. The vertical line denotes the switch to the new relevance algorithm, Best Match, which is followed by a significant and steady increase in usage. Note that the 1% usage rate on the *y*-axis represents about 30,000 queries on an average work day.

We have observed that the CTRs of relevance search using the new Best Match algorithm have continued to increase since June. Moreover, there is a rapid growth in the overall usage of the relevance sort option. As shown in Fig 3, usage of the relevance sort is steadily increasing with a faster increase since Best Match has been deployed. From June 2017 to April 2018, the overall usage of relevance search has increased from 7.5% to 12% (a 60% increase) of all PubMed queries.

### The new ranking system is highly scalable

The proposed system has been optimized for throughput (see S9 Text) so that it is able to scale up and exceed the real-world throughput requirement of PubMed searches, approximately 200 queries per second. At maximum, our system is able to process approximately 700 queries per second at an average of approximately 70 ms per query as we run 100 threads in parallel.

### Best practices for using Best Match

Generally speaking, PubMed queries can be categorized in two broad classes: navigational versus informational. Navigational searches, also referred to as known-item searches, are ones in which the search intent is to find a specific article or set of articles (e.g., a search with an article title or author name). On the other hand, informational searches seek to find and/or explore articles satisfying information needs on a given topic (e.g., using a query like "HIV DVT" to gather evidence of deep vein thrombosis related to HIV). In this regard, Best Match is more appropriate for the latter use cases, for which the most relevant set of results are desired, and is therefore complementary to the traditional Most Recent sort order in PubMed.

As mentioned earlier, to familiarize our users with the newly developed Best Match search, a banner is displayed as shown in Fig 2 when appropriate. That is, each time a search is run under the default "Most Recent" sort order and the query is found to be informational by the Field Sensor [28], the Best Match banner will be triggered. However, in order to minimize any potential disruption of usual PubMed searches, it is not triggered if the query returns less than 20 results or if other results are displayed, such as those from our spell checker. As a result of these rules, currently Best Match banners are only triggered for about 35% of the total PubMed queries, though topical searches generally account for half of total searches in PubMed.

Finally, as we know different information needs may be better fulfilled by different sort orders [29], we have improved PubMed’s usability by making it simple for our users to choose and switch between the two sort orders. In particular, we have implemented and added a two-part toggle at the top right in the search results page, which allows users to conveniently change between the two most used search modes, "Most Recent" and "Best Match." When users switch the sort order, using this new toggle function or the traditional "Sort By" drop-down menu, it is saved automatically so that all further searches will run using the new order. Because of the recent success of "Best Match" in PubMed, this mode is now being tested as the default sort order in the newly developed PubMed Labs (www.pubmed.gov/labs) system, in which search results are further accompanied with rich snippets.

## Discussion

As mentioned, there is unfortunately no existing dataset that meets the need for a machine-learning–based retrieval system for PubMed, and it is not possible to manually curate a large-scale relevance data set. Hence, we adopted a common industry practice for assembling a gold-standard training dataset through the extraction of click-through data in search logs as pseudorelevance [30–34].

There are several known issues with this method. First, in our logs, the number of searches using relevance sort is still modest at present. Over the last year, we were able to collect some data (about 46,000 queries) to train a ranking model. To this end, we need queries that are frequent and with explicit user actions so that we have relevance estimation of articles with regards to these queries. In 2016, with about 150,000 queries run under Best Match per month, only hundreds of them met the threshold to build a gold standard (see S3 Text for details on the filters and threshold used and S4 Text for details on the gold standard creation). But, as relevance search gains popularity in PubMed, we will soon be able to collect several thousands of recurrent queries every few months to better train the ranker over time.

Second, when users click a result or request the full text of an article, they often do not explore the entire set of search results. Hence, potentially relevant documents may be missed in the gold standard or considered as irrelevant. Conversely, when an article is clicked, it could still be irrelevant to the user information need.

Third, there is a potential bias in the fact that we do not account for the position in which clicked documents were ranked. In other words, if a document is clicked at the 10th position, it should, in theory, have more weight in training than the one at the first position because the top document is naturally more likely to be clicked. We are currently experimenting with ways to account for this particular factor during the creation of training data.

In summary, this paper presents the latest major improvement in PubMed for relevance search. We used a state-of-the-art information retrieval technique, adapted it to the biological domain (e.g., by creating training data and ranking features specific to the scientific literature), and scaled it to meet the throughput requirement of PubMed with millions of searches each day. Specifically, we developed an end-to-end pipeline based on an open source search platform (Solr) and an advanced machine-learning algorithm (LambdaMART) for optimizing the quality of the top-ranked results. We described in detail what features ("signals") we selected for the machine-learning algorithm, how they were evaluated, and in what way they contribute to the final ranking results. This paper also demonstrates the whole process and steps in adopting state-of-the-art research findings into a real-world application such as offline versus online evaluation, scalability test, usage analysis, etc.

Overall, the new Best Match algorithm shows a significant improvement in finding relevant information over the default time order in PubMed. It has also resulted in an increased usage of relevance search over time, which allows us to accumulate more relevance data for iteratively improving our machine-learning–based ranker.

We have also noticed that in the last few years, the IR community has started developing and experimenting with new retrieval methods for document ranking using the latest deep-learning techniques. While early results (including our own) are promising [35–41], more work is warranted with regards to retrieval quality, robustness, and scalability for adoption into real-world applications such as PubMed.

Finally, it is important to note that we design and build our methods based on our users and their search behaviors. Therefore, we encourage them to try this new relevance search and provide input so that they can help us continue to improve the ranking method.

## Supporting information

S1 Glossary List of abbreviations and definitions. (PDF)

S1 Text First stage ranking by BM25. (PDF)

S2 Text Second stage ranking by L2R. (PDF)

S3 Text Search log data. (PDF)

S4 Text Generating gold-standard relevance data. (PDF)

S5 Text Evaluation metrics. (PDF)

S6 Text Improved ranking quality in offline benchmarking evaluation. (PDF)

S7 Text Feature representation. (PDF)

S8 Text Feature contribution. (PDF)

S9 Text System setup and optimization. (PDF)

S1 Fig Average click through rate at rank 20 for queries occurring less than 1,000 times. The observed overall average CTR@20 of near 0.4 appears to be strongly influenced by unique queries. The chart is cut at 1,000, but only a minimal number of queries occur more than a thousand times over a year.
(TIF)

S2 Fig Offline evaluation of the new relevance algorithm against the silver standard extracted from the search logs. Precision-recall curves are plotted after the first step (green) and the second (blue) accordingly. A much higher precision is achieved after the second re-ranking step, especially for the top ranked results.
(TIF)

S3 Fig Impact of feature ablation on overall ranking quality (measured by NDCG@20 scores). (TIF)

## Abbreviations

BM25: Okapi Best-Matching algorithm
CTR: click-through rate
D: document
DVT: deep vein thrombosis
IR: information retrieval
L2R: learning to rank
NCBI: National Center for Biotechnology Information
NDCG: Normalized Discounted Cumulative Gain
NIH: National Institutes of Health
NLM: National Library of Medicine
Q: query
QD: query–document relationship
TF–IDF: term frequency–inverse document frequency
TREC: Text REtrieval Conference
