# Supplementary material for: Best Match: New relevance search for PubMed
Source: PLoS Biol. 2018 Aug 28;16(8):e2005343. doi: 10.1371/journal.pbio.2005343 (PMC6112631; doi:10.1371/journal.pbio.2005343)
Supplement: S1 Glossary — (PDF) [file pbio.2005343.s001.pdf]

S1 Glossary

List of abbreviations and definitions

A/B testing A method to compare two or more variants (A and B), that are identical except for one variation being tested. This intuition behind this approach is similar to case-control studies in biomedicine.

Best Match The name of the new relevance sort option used in PubMed and described in this article.

BM25 Also referred to as Okapi BM25, is a classic probabilistic term weighting model for document ranking. It is defined in S1 Text and used as the first-step ranking function in our overall method.

CTR Short for Click Through Rate, a metric for the rate at which a specific online presentation (e.g. a web advertisement) is clicked. It is detailed in S5 Text.

Field A document field is a part of a PubMed record, e.g. title, abstract or author names.

Gold Standard A set of training examples, considered as ground truth required for training machine learning models.

Held-out test data An important method for ensuring that the metrics for a model do not reflect over-fitting on training data is to have a hold-out set for testing, consisting of data that was not used during training.

IR Short for information retrieval: finding information matching a user need. In this article, it refers to retrieving PubMed articles relevant to a user query.

L2R Short for Learning-to-rank, a class of machine learning methods that focuses on optimally ranking a set of items. In this work, L2R is used for document ranking.

LambdaMART A highly effective and efficient learning-to-rank algorithm we applied for re-ranking results in the second step of our overall method, detailed in S2 Text.

Machine Learning A field of computer science focusing on teaching computers to learn from training data without explicit rule programming.

NDCG Short for Normalized Discounted Cumulative Gain, a standard metric to evaluate ranked lists against a gold standard, detailed in S5 Text.

TF-IDF A traditional method to measure the relative importance of a query term to a given document in the entire document collection based on two factors (a) TF: term frequency - how often a query term occurs in a given document and (b) IDF: inverse document frequency - whether the term is rare or common across all documents). It is defined in S1 Text.
