# Supplementary material for: Best Match: New relevance search for PubMed
Source: PLoS Biol. 2018 Aug 28;16(8):e2005343. doi: 10.1371/journal.pbio.2005343 (PMC6112631; doi:10.1371/journal.pbio.2005343)
Supplement: S1 Text — (PDF) [file pbio.2005343.s002.pdf]

S1 Text

First stage ranking by BM25

BM25 with extension to multiple weighted Fields (BM25F) [1] is a frequentist scoring function. As mentioned before, each document in PubMed consists of a set of data fields (i.e. title, abstract, etc.) and within each field there is a bag of words or phrases — or MeSH concepts identifiers for the MeSH field. During indexing, the term frequencies of all words within document fields are collected and stored. During retrieval, these frequencies are used to calculate a prior score for PubMed documents matching the terms in the user's query. The prior relevance of a document to a single term query depends on the following:

- $tf(t)$: the local weight of a term t, or term frequency. It is the sum of its frequencies over all the fields of a document, weighted by the respective field weights w and averaged over lengths l.

$$tf(t) = \sum_{f \in fields} \frac{occ(t,f) \times w(f)}{l(f)}, \tag{1}$$

  where $occ(t,f)$ is the count of t in the field f.

- $idf(t)$: the global weight of a term t, or inverse document frequency. It is:

$$idf(t) = 1 + ln\left(\frac{N}{n(t)+1}\right), \tag{2}$$

  where N is the total number of documents and $n(t)$ is the size (count) of its subset of documents containing the term t.

- $w(f)$: the weight of a field f. This weight allows one to give more importance to a certain information stream, e.g. currently, matches in the title and MeSH fields are multiplied by a weight of 5 while other field are not weighted.

- $l(f)$: the length of a field f. The sum of all field lengths of a document is the document length.

The computation of BM25F requires combining field lengths and weights into a document length dl and its average in the corpus:

$$dl = \sum_{f \in fields} l(f) \times w(f), \tag{3}$$

$$avg(dl) = average\ of\ dl\ across\ alldocuments. \tag{4}$$

Then, the prior relevance score associated with a document d for a query q is defined as

$$BM25F(d,q) = \sum_{t \in q \cap d} \frac{tf(t)}{k_1\left((1-b) + b\frac{dl}{avg(dl)}\right) + tf(t)} \times idf(t) \tag{5}$$

where k_1 and b are hyper parameters empirically set to 1.2 and 0.75 respectively, in PubMed. We also experimented with divergence from randomness (DfR) [2] for the first stage ranking and combining DfR with BM25. No improvement was observed and the results can be found at our GitHub repository.

References

[1] S. Robertson and H. Zaragoza. The probabilistic relevance framework: BM25 and beyond. *Found. Trends Inf. Retr.*, 3(4):333–389, 2009.

[2] G. Amati, and C.J. Van Rijsbergen. Probabilistic models of information retrieval based on measuring the divergence from randomness. *ACM Transactions on Information Systems (TOIS)*, 20(4):357–389, 2002.
