# Supplementary material for: Best Match: New relevance search for PubMed
Source: PLoS Biol. 2018 Aug 28;16(8):e2005343. doi: 10.1371/journal.pbio.2005343 (PMC6112631; doi:10.1371/journal.pbio.2005343)
Supplement: S2 Text — (PDF) [file pbio.2005343.s003.pdf]

S2 Text

Second stage ranking by L2R

Once the prior relevance is calculated, it is used to pre-rank documents before the L2R layer in the second stage. BM25F is a robust metric that yields a good prior ranking such that there is no need to process all returned documents through L2R as this would be too expensive computationally. The reason we selected LambdaMART over other L2R approaches is that it is fast enough for a production environment, especially compared to list-wise L2R approaches which evaluate and compare entire lists of results [1, 2, 3]. Instead, LambdaMART is a pair-wise L2R framework, based on gradient boosted regression trees, that optimizes a list-wise cost metric. In our case, we chose NDCG@20 as described in S5 Text.

Suppose the training dataset consists of a set of PubMed queries $Q = \{q_1, ..., q_N\}$, and each query matches a set of citations $D = \{d_1, ..., d_K\}$ with their respective relevance labels $L = \{l_., ..., l_K\}$ (see S4 Text for their calculation). Each document d_i is a feature vector in R^{179} – we have 179 features. The objective of the ranker is to find the function $f : R^{179} \Rightarrow \mathbb{R}$ such that documents ordered according to their predicted scores $S = \{s_1 = f(d_1), ..., s_K = f(d_K)\}$ in descending order, with the resulting ranking the closest to that of L, i.e. to the gold standard.

The objective of LambdaMART is to determine how to score documents to minimize the distance, based on the selected cost metric, between the predicted ranking and the gold standard. Specifically, it focuses on learning how documents should be ranked. Say LambdaMART is comparing two documents d_u, d_v with relevance labels $l_u > l_v$. The objective function [4] is defined as:

$$O_{uv} = -o_{uv} + log(1 + e^{o_{uv}}), \tag{1}$$

where $o_{uv} = s_u - s_v$. This idea was first introduced in RankNet [5], which optimizes for the number of pairwise errors in ranking. Later on, LambdaRank, a precursor to LambdaMART, was based on the empirical observation that weighting the derivative of this cost by the change in NDCG obtained by swapping d_u and d_v, results in a system that optimizes for NDCG. Therefore, in LambdaMART, a weighted derivative of the objective function is then used to identify whether documents should be swapped in order to maximize the $NDCG@k$ score (the ranking quality at rank k):

$$\lambda_{uv} = |\Delta NDCG@k(s_u, s_v)| \times \frac{\delta O_{uv}}{\delta o_{uv}}, \tag{2}$$

$$\frac{\delta O_{uv}}{\delta o_{uv}} = \frac{\delta O_{uv}}{\delta s_u} = -\frac{1}{1 + e^{o_{uv}}}, \tag{3}$$

$$\Delta NDCG@k(s_u, s_v) = N(2^{l_u} - 2^{l_v}) \left(\frac{1}{log(1 + t(d_u))} - \frac{1}{log(1 + t(d_v))} \right), \tag{4}$$

where $t(\cdot)$ is the rank of a document. Although many different metrics could be optimized for ranking, NDCG yielded the best ranking – quality results in our experiments. By integrating NDCG when calculating the λ's, LambdaMART is able to learn both the distance each document score should be moved as well as the direction. A gradient is then calculated for each document d_u by summing the gradients associated with all pairs of documents (d_u, d_v) for a given query:

$$\lambda_u = \sum_{v \in (d_u, d_v): l_u \neq l_v} \lambda_{uv}. \tag{5}$$

At each training iteration, LambdaMART builds a gradient boosted regression tree that guides ranking by promoting or demoting documents based on the direction and distance provided by their λ-gradient. The combination of all weak learners (boosted trees) results in a robust and efficient ranking model.

References

[1] M. Taylor, J. Guiver, S. Robertson, and T. Minka. Softrank: optimizing non-smooth rank metrics. In *Proceedings of the 2008 International Conference on Web Search and Data Mining*, pages 77–86. ACM, 2008.

[2] J. Xu and H. Li. Adarank: a boosting algorithm for information retrieval. In *Proceedings of the 30th annual international ACM SIGIR conference on Research and development in information retrieval*, pages 391–398. ACM, 2007.

[3] F. Xia, T.-Y. Liu, J. Wang, W. Zhang, and H. Li. Listwise approach to learning to rank: theory and algorithm. In *Proceedings of the 25th international conference on Machine learning*, pages 1192–1199. ACM, 2008.

[4] K. M. Svore, M. N. Volkovs, and C. JC Burges. Learning to rank with multiple objective functions. In *Proceedings of the 20th international conference on World wide web*, pages 367–376. ACM, 2011.

[5] C. Burges, T. Shaked, E. Renshaw, A. Lazier, M. Deeds, N. Hamilton, and G. Hullender. Learning to rank using gradient descent. In *Proceedings of the 22nd international conference on Machine learning*, pages 89–96. ACM, 2005.
