# Supplementary material for: Best Match: New relevance search for PubMed
Source: PLoS Biol. 2018 Aug 28;16(8):e2005343. doi: 10.1371/journal.pbio.2005343 (PMC6112631; doi:10.1371/journal.pbio.2005343)
Supplement: S3 Text — (PDF) [file pbio.2005343.s004.pdf]

S3 Text

Search log data

In order to train LambdaMART and test its effectiveness, gold-standard query-document pairs are required. Given the lack of real-world datasets for biomedical information retrieval, we created a training dataset from PubMed search logs. We used anonymous user queries as well as subsequent clicks on returned articles with the assumption that user actions are an approximation for relevance in PubMed. Although we cannot share the log data per NIH/NLM/NCBI security and privacy policy (`https://www.nlm.nih.gov/privacy.html`), we describe how it was generated and its overall statistics in detail below. We first collected 614 days' worth of query logs in an aggregated and anonymized form. Queries that used specific search facets, had less than 20 results, or used Boolean operators were removed, resulting in approximately 2,000,000 queries. For each query and its returned results, we recorded two types of user actions: a) clicks to read an abstract and b) clicks to request the full text. We then discarded infrequent queries (appearing less than 3 times) in the entire dataset. This threshold was set empirically to remove noisy queries, as previously suggested [1]. Next, all duplicate queries were then merged and their associated click data were accumulated, which resulted in approximately 67,000 unique queries. Before merging, 44% of the queries in this dataset initially appeared three times, 20% appeared four times and 11% appeared five times. A large majority of the dataset is thus made of rare queries occurring 5 times or less in almost two years' worth of logs. For efficient training, we further removed 21,000 queries with less than 20 positive articles (clicked by the users). As a result, we have a set of 46,000 unique queries, each of which is associated with at least 20 positive PMIDs (23 on average). The queries consist of 2.30 terms on average (from 1 to 18) and a median length of 2. Some example queries are: "pancreatic neuroendocrine tumor", "systematic review type b dissection", and "paeoniflorin".

References

[1] M. Shokouhi. Learning to personalize query auto-completion. In *Proceedings of the 36th international ACM SIGIR conference on Research and development in information retrieval*, pages 103-112. ACM, 2013.
