# Supplementary material for: Best Match: New relevance search for PubMed
Source: PLoS Biol. 2018 Aug 28;16(8):e2005343. doi: 10.1371/journal.pbio.2005343 (PMC6112631; doi:10.1371/journal.pbio.2005343)
Supplement: S4 Text — (PDF) [file pbio.2005343.s005.pdf]

S4 Text

Generating gold-standard relevance data

With two types of clicks (abstract and full-text clicks), we computed relevance levels for each of the clicked documents associated with those 46,000 queries. Let us denote a document by d and a query by q. $a(d,q)$ is the number of abstract requests for d after q. $f(d,q)$ is the number of full text requests for d after q. FT represents the subset of articles in the corpus for which the full text is available. $1_{FT}(d)$ is an indicator function such that

$$1_{FT}(d) := \begin{cases} 1 \; if \;\; d \in FT, \;\; full \; text \; is \; available, \\ 0 \; if \;\; d \notin FT, \;\; full \; text \; is \; not \; available. \end{cases} \tag{1}$$

The relevance score of d with regard to q is calculated as follows:

$$relevance\_level(d,q) = \mu \cdot a(d,q) + (1-\mu) \cdot f(d,q) + \frac{a(d,q)}{\lambda} \cdot (1 - 1_{FT}(d)). \tag{2}$$

Where $\mu \in (0,1]$ is the weight of abstract versus full text clicks and $\lambda \in \mathbb{R}^+$ is the boost for papers where full-text links do not exist in PubMed. This boost factor is important because two articles with the same number of abstract clicks and zero full-text clicks should not be scored equally if the full text is available for one but not for the other. μ and λ were empirically determined in our experiments. After this step, for each query all of its clicked documents (about 34 on average) are sorted by their computed relevance levels, respectively.

As detailed in [1], relevance labels can be either binary or non-binary depending on the application and available data. Using more than binary labels (e.g. “perfect”, “excellent”, “good”, “fair”, “bad”) is usually more challenging for the ranker but allows to better learn how to optimize the top results in the list. Therefore, to aim for the best results in the top ranks, articles that appear with the ten highest relevance levels are given a score of 12 to 3, the following 10 articles a score of 2, and the remaining relevant documents a score of 1. All other documents without relevance levels (i.e. irrelevant articles) receive a score of 0. The set of 46,000 queries were randomly split into 70% for training and 30% for testing. On average, each query in the training and test datasets is associated with 23 articles with a non-zero score.

References

[1] O. Chapelle and Y. Chang. Yahoo! learning to rank challenge overview. In *Yahoo! Learning to Rank Challenge*, pages 1–24, 2011.
