# Supplementary material for: Best Match: New relevance search for PubMed
Source: PLoS Biol. 2018 Aug 28;16(8):e2005343. doi: 10.1371/journal.pbio.2005343 (PMC6112631; doi:10.1371/journal.pbio.2005343)
Supplement: S5 Text — (PDF) [file pbio.2005343.s006.pdf]

S5 Text

Evaluation metrics

Normalized Discounted Cumulative Gain (NDCG) [1] is a standard ranking metric in the information retrieval research area that penalizes placement of relevant documents at lower rankers, computed as follows:

$$NDCG@k = \frac{DCG@k}{IDCG@k}, \tag{1}$$

$$DCG@k = \sum_{i=1}^{k} \frac{2^{l_i} - 1}{log_2(i+1)} \tag{2}$$

where $IDCG@k$ is the ideal Discounted Cumulative Gain ($DCG@k$), i.e. for the gold standard relevance order. l_i is the relevance of the document at rank i in the list to be evaluated. Since PubMed returns up to 20 results in the first page, we chose $k = 20$ and aimed to optimize NDCG@20 scores during model training.

In addition to using the offline metrics for measuring relevance, we evaluated our proposed relevance search in an online mode with real PubMed users. To this end, we used click-through rate (CTR) as defined below:

$$CTR@k = \frac{|queries\ with\ a\ click\ on\ rank\ k\ or\ lower|}{|eligible\ queries|}, \tag{3}$$

where eligible queries are queries returning more than 1 document. CTR is a widely-used measure for evaluating the success of new features for a particular website (e.g. common in online advertising) in so-called A/B testing, a controlled experiment with two (or more) variants. We employed it to determine whether our new relevance sort algorithm results in improved search experience to our users in practice. In order to obtain a finer-grained understanding of our system performance in top ranked results, we calculated CTR scores for the top 20, 10, 5, and 3 results.

References

[1] K. Järvelin and J. Kekäläinen.Cumulated gain-based evaluation of IR techniques.*ACM Transactions on Information Systems (TOIS)*, 20(4):422–446, 2002.
