# Supplementary material for: Best Match: New relevance search for PubMed
Source: PLoS Biol. 2018 Aug 28;16(8):e2005343. doi: 10.1371/journal.pbio.2005343 (PMC6112631; doi:10.1371/journal.pbio.2005343)
Supplement: S6 Text — (PDF) [file pbio.2005343.s007.pdf]

# S6 Text

## Improved ranking quality in offline benchmarking evaluation

Using the user-click information from PubMed search logs as the (pseudo-)gold standard (or silver standard) for document relevance, we first evaluated our two-step ranking system using NDCG, a standard measure for ranking quality (see details in S5 Text), for assessing ranking quality and comparing scores after each step. The extracted click-through dataset contains 46,000 unique queries, which were randomly split into a training set of 70% and test set of 30%. And on average, each query in the two datasets is associated with 23 relevant articles and 477 irrelevant ones (500 in total). We chose 500 empirically in this work because overall it represents a good coverage of those clicked articles (68% or 23/34 articles) previously collected from the search logs. While a higher coverage is desired, using more documents would not only make it less efficient but also more challenging for the L2R algorithm to perform well. This is because a larger proportion of irrelevant articles would be included in the dataset. For example, we observed that increasing N from 500 to 1,000 would result in only three more relevant articles (from 23 to 26) but more than twice as many irrelevant articles (from 477 to 974), thus making it much more difficult for the algorithm to learn an effective model. On the other hand, reducing N to 250 would decrease the number of relevant documents retrieved by 30% (16 instead of 23) and a threshold at 100 would yield only 12 relevant documents.

As described, the first-stage ranker is the classic BM25F method followed by LambdaMART. Our system achieved 0.15 in NDCG@20, after ranking by BM25 in step 1 and 0.48 by re-ranking in step 2, respectively. This result shows that LambdaMART is able to learn from the "ground truth" in the training set with the set of proposed features. Meanwhile, 0.48 in NDCG@20 also suggests that a perfect ranking (i.e. all relevant articles are ranked by L2R in the exact same order as what is in the gold standard) is highly challenging [1]. This is largely due to the imbalance of relevant vs. irrelevant items in the dataset (23 vs. 477): a random order would yield an NDCG@20 score of 0.025. Furthermore, we used traditional information retrieval metrics, precision and recall, to evaluate our approach. The precision is the fraction of relevant documents among the retrieved documents and recall is the fraction of relevant documents that have been retrieved over the total amount of relevant documents for a query. We computed precision-recall curves based on the corresponding results of each query in the test set. It is clear from S2 Fig that by re-ordering the top 500 search results in step 1, the second ranker consistently achieves a much higher precision at each rank position when compared with results from the first step using BM25 only. The distribution of NDCG@20 scores for both layers and the relative improvement or deterioration for the 46,000 queries are also provided in the GitHub repository.

## References

[1] Y. Wang, L. Wang, Y. Li, D. He, W. Chen, and T. Liu. A theoretical analysis of NDCG ranking measures. In *Proceedings of the 26th Annual Conference on Learning Theory (COLT 2013)*, 2013.
