# Supplementary material for: Best Match: New relevance search for PubMed
Source: PLoS Biol. 2018 Aug 28;16(8):e2005343. doi: 10.1371/journal.pbio.2005343 (PMC6112631; doi:10.1371/journal.pbio.2005343)
Supplement: S7 Text — (PDF) [file pbio.2005343.s008.pdf]

S7 Text

Feature representation

Note that some of the features have numerical values (e.g. BM25 score) while others are categorical variables. Especially, for the latter to be interpreted as categorical features by the machine learning algorithm, language is one-hot encoded (one 1 for the main language and 59 0s for other languages); publication types are n-hot encoded (multiple 1s and 0s). As a result, there exist over 150 features effectively in our system. The complete list of features is provided in Fig 1b.
