# Supplementary material for: Best Match: New relevance search for PubMed
Source: PLoS Biol. 2018 Aug 28;16(8):e2005343. doi: 10.1371/journal.pbio.2005343 (PMC6112631; doi:10.1371/journal.pbio.2005343)
Supplement: S8 Text — (PDF) [file pbio.2005343.s009.pdf]

S8 Text

Feature contribution

The LambdaMART algorithm does not directly indicate the importance of each feature used in the final model, which consists of a forest of gradient boosted decision trees. The common approach to estimate feature contributions is to perform feature ablation studies to identify (groups of) features that, when removed, lead to the highest drop in performance [1]. S3 Fig shows the performance associated with the removal of individual feature types, where categorical features are treated as one (e.g., publication type). As can be seen, D (especially publication year F1 and click information F2-3) and QD (especially BM25 F143) features are the ones that contribute the most to the loss in NDCG scores when removed.

As further detailed in S1 Table, the removal of the group of D or QD features as a whole leads to a significant drop in NDCG@20 scores (the same as using BM25F only). The QD features capture the relevance between documents and queries while the D features are independent measures of documents only. They each play significant roles in ranking and when combined, the NDCG scores double, suggesting that these two groups of features are complementary to each other.

S1 Table.

Feature ablation study

	Setup	NDCG@20
	Using BM25F only	0.15
	Using L2R with all features	0.48
1-category ablation	L2R with Q features removed	0.47
	L2R with D features removed	0.23
	L2R with QD features removed	0.26
2-category ablation	L2R with D features only	0.25
	L2R with Q features only	0.15
	L2R with QD features only	0.22

References

[1] M. Richardson, A. Prakash, and E. Brill. Beyond pagerank: machine learning for static ranking. In *Proceedings of the 15th international conference on World Wide Web*, pages 707–715. ACM, 2006.
