# Supplementary material for: Best Match: New relevance search for PubMed
Source: PLoS Biol. 2018 Aug 28;16(8):e2005343. doi: 10.1371/journal.pbio.2005343 (PMC6112631; doi:10.1371/journal.pbio.2005343)
Supplement: S9 Text — (PDF) [file pbio.2005343.s010.pdf]

S9 Text

System setup and optimization

The Best Match algorithm is built based on Solr (`http://lucene.apache.org/solr/`) as Solr is open source and can be customized. BM25F is a built-in ranking function in Solr and the LamdaMART algorithm became a native plugin for Solr v6.6 in June 2017. Specifically, Solr is deployed on 8 nodes where each contains 16 CPUs, 30GB of RAM and 320GB SSD storage. The entire search index is randomly partitioned among four shards, each of which consists of two identical nodes for better scalability, security and load balancing. We also optimized the complexity of feature calculation algorithms for the top 500 documents returned by BM25, as this was the most computationally expensive step. In particular, one-hot and n-hot encoded features (language and publication types, respectively) were the most computationally intensive. Therefore, we improved their implementation so that the computational complexity becomes $\mathcal{O}(1)$ instead of $\mathcal{O}(e)$ for these features, where e is the encoding dimension. To save time, we also make use of parallelization, by processing one request per thread, with 100 threads.
