# Supplementary figures and images for: Best Match: New relevance search for PubMed
Source: PLoS Biol. 2018 Aug 28;16(8):e2005343. doi: 10.1371/journal.pbio.2005343 (PMC6112631; doi:10.1371/journal.pbio.2005343)

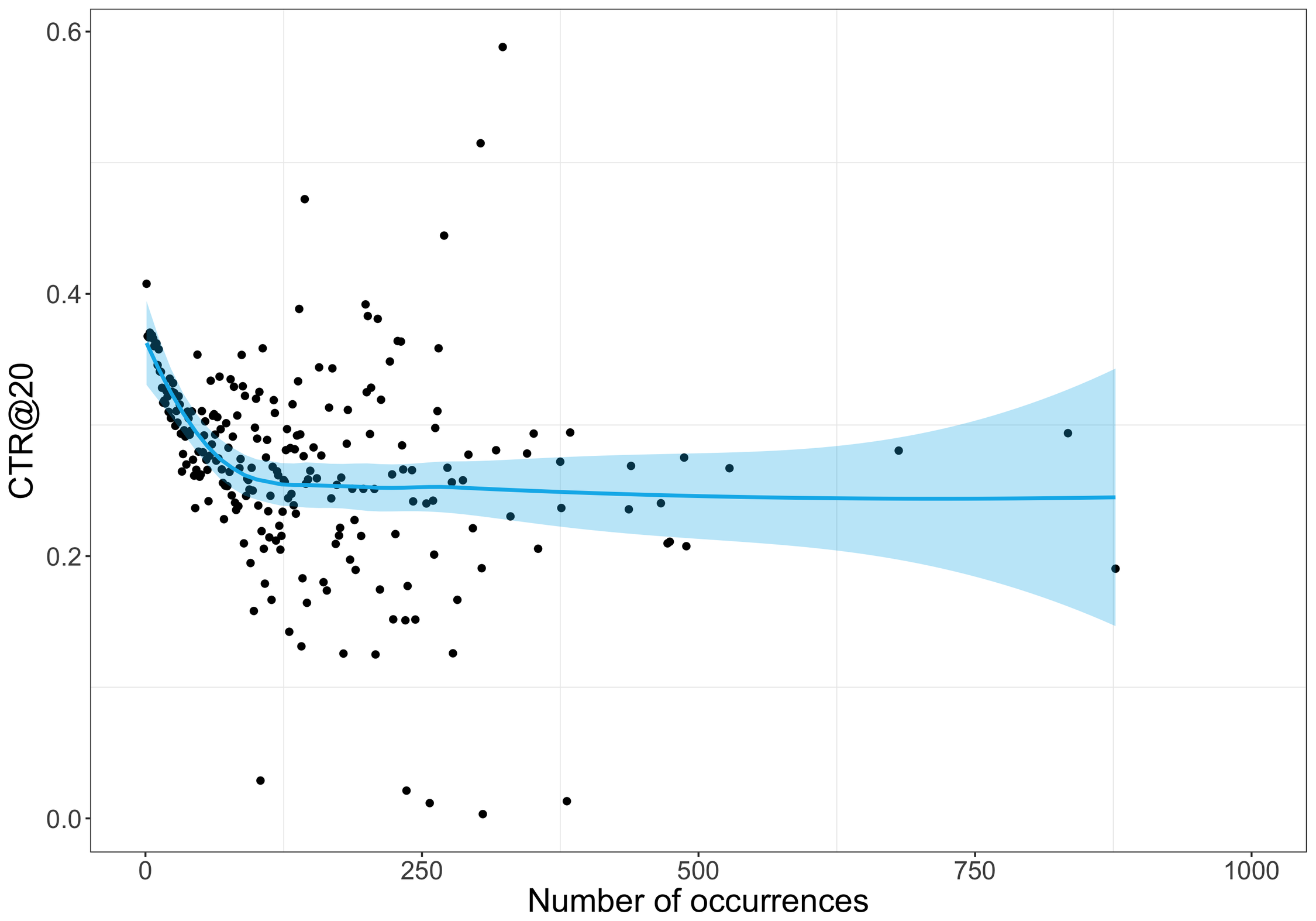

Supplement: S1 Fig — The observed overall average CTR@20 of near 0.4 appears to be strongly influenced by unique queries. The chart is cut at 1,000, but only a minimal number of queries occur more than a thousand times over a year. (TIF) [file pbio.2005343.s011.tif]

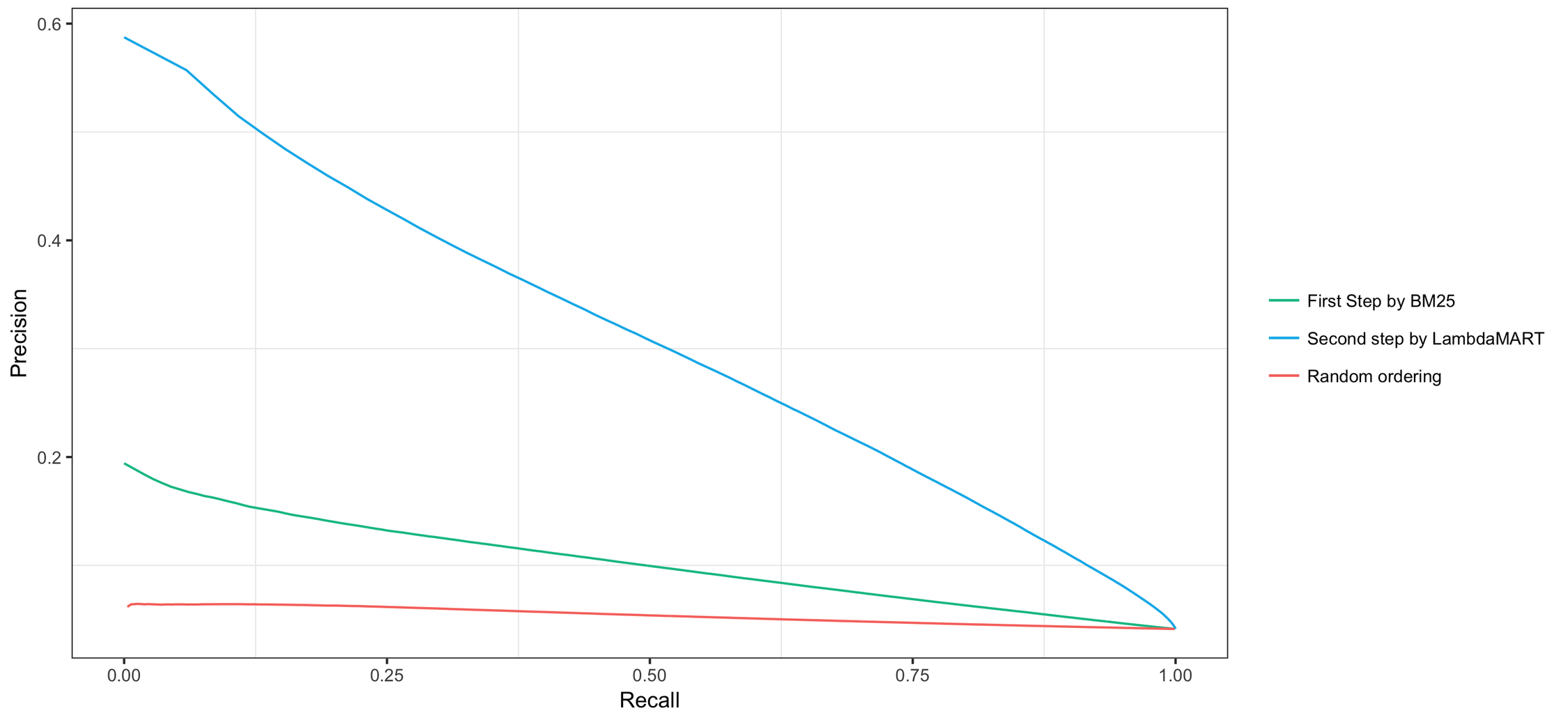

Supplement: S2 Fig — Precision-recall curves are plotted after the first step (green) and the second (blue) accordingly. A much higher precision is achieved after the second re-ranking step, especially for the top ranked results. (TIF) [file pbio.2005343.s012.tif]

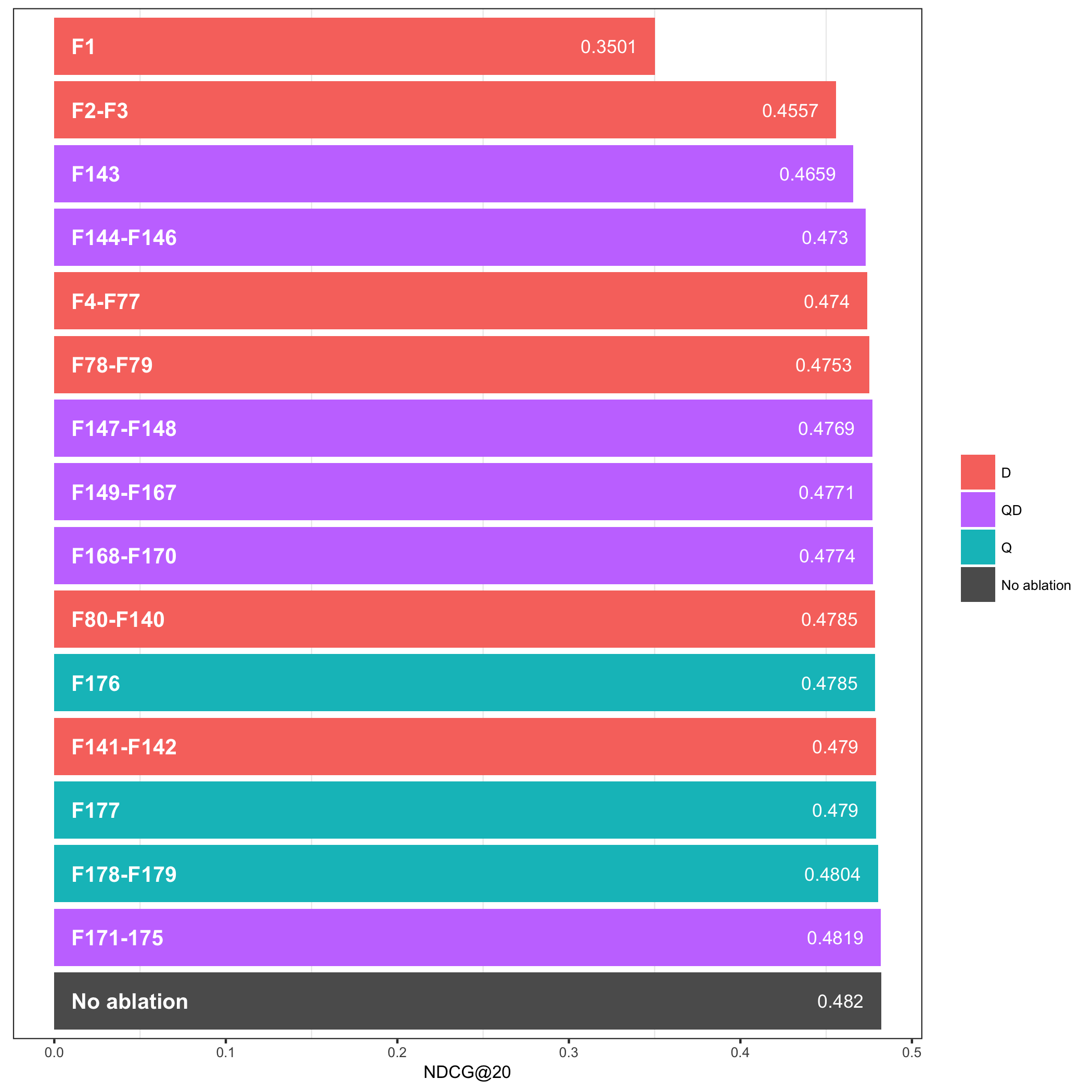

Supplement: S3 Fig — (TIF) [file pbio.2005343.s013.tif]
